# Minnesota smokers’ perceived helpfulness of 2009 federal tobacco tax increase in assisting smoking cessation: a prospective cohort study

**DOI:** 10.1186/1471-2458-13-965

**Published:** 2013-10-18

**Authors:** Kelvin Choi, Raymond G Boyle

**Affiliations:** 1 Division of Epidemiology and Community Health, University of Minnesota, 1300 South Second Street # 300, Minneapolis, MN 55454, USA; 2 ClearWay Minnesota SM, 8011 34th Ave S # 400, Minneapolis, MN 55425, USA

**Keywords:** Smoking, Taxation, Cessation, Perception

## Abstract

**Background:**

The cost of cigarettes has been cited as a motivating factor for smokers to quit smoking, and a cigarette tax increase is an effective way to increase the cost of cigarettes. Scholars have suggested that smokers may see cigarette tax increases as commitment devices to help them quit smoking. Little is known about whether smokers actually think cigarette tax increases help them quit, and whether this perception predicts subsequent smoking cessation behaviors. We used data from the Minnesota Adult Tobacco Survey Cohort Study collected after the 2009 federal tobacco tax increase to answer these questions.

**Methods:**

In 2009, 727 smokers were asked whether they thought the federal tobacco tax increase helped them to: (1) think about quitting, (2) cut down on cigarettes, and (3) make a quit attempt. We also collected data on demographics, number of cigarette price-minimizing strategies used, and cigarette consumption. In 2010, we assessed if these smokers had made a quit attempt, had cut down on their cigarette consumption, and had stopped smoking. Logistic regression models were used to assess the characteristics associated with the perceptions that the tax increase was helpful in assisting smoking cessation, and the association between these perceptions in 2009 and cessation behaviors in 2010.

**Results:**

Overall, 65% of the sample thought that the 2009 tax increase helped them think about quitting, 47% thought it helped them cut down on cigarettes, and 29% thought it helped them make a quit attempt. Lower education, lower income, lower cigarette consumption, and using more cigarette price-minimizing strategies were associated with the perceptions that the tax increase was helpful in assisting smoking cessation (p < 0.05). Smokers who perceived the tax increase as helpful in assisting smoking cessation were more likely than those who did not perceive the tax increase as helpful to report making a quit attempt in 2010 (p < 0.05).

**Conclusions:**

A significant proportion of smokers in our sample thought the 2009 federal tobacco tax increase was helpful in assisting smoking cessation, particularly among smokers of lower socio-economic status. Health communication interventions to promote cigarette tax increases as an opportunity for smoking cessation may further assist quit attempts.

## Background

Smoking remains a leading public health problem in the US. About 1 in 5 US adults is a smoker [1], and reducing cigarette smoking by adults is a national objective [2]. Assisting smokers in quitting is considered an important strategy to reduce the prevalence of smoking. Because smoking cessation is beneficial to health regardless of age [3], reducing the prevalence of smoking will result in a reduction in health care expenditures and smoking-associated morbidity and mortality. National data showed that 69% of adult smokers are interested in quitting smoking [4]. However, given the challenge of the addiction, even though 52% of smokers have tried to quit smoking in the past year, only 6% successfully stop smoking [4]. It usually requires multiple attempts for smokers to be successful in quitting smoking [3], and it is important to help smokers keep trying.

Increasing costs of cigarettes has been cited by smokers as an important factor in their intentions to quit smoking [5]. A study from Australia found that 60% of smokers would seriously consider quitting smoking if the cost of their usual cigarette brand increased by 33%, and 88% of them would seriously consider quitting if the cost increased by 67% [6]. Cigarette taxes are an effective way to increase the cost of cigarettes. After a 10% cigarette tax increase in New Zealand twice as many smokers reported cost as a reason for attempting to quit smoking (25.5% in 2009 vs. 55.6% in 2010) [7]. Previous research has shown that cigarette tax increases are associated with increases in smoking cessation and/or reducing cigarette consumption [8], particularly among lower-income smokers [9,10]. One mechanism by which cigarette tax increases may influence smoking behaviors is to reduce the affordability of cigarettes. As cigarette taxes increase the prices of cigarettes, smokers have to spend more money on cigarettes, or modify their smoking behaviors to control their cigarette expenditures. Another potential mechanism is cigarette tax increases may act as a commitment device to smoking cessation. Commitment devices are defined as strategies that reduce the utility of smoking to enable smokers to commit to cessation [11,12], potentially through supporting smokers to act on their intention to quit smoking. Under this framework, smokers change their smoking behaviors out of their desire (supported by the commitment device), instead of changing their smoking behaviors out of financial necessity.

On April 1, 2009, a federal tobacco excise tax increase raised the price of cigarettes by 61 cents per pack, which increased the average retail price for a pack of cigarette in the US to $4.35 [13]. Excise taxes for other tobacco products (e.g., smokeless tobacco, cigars) were also increased to fund the Children’s Health Insurance Program. While a previous study showed that call volumes to quitline increased by 23.5% in 16 states after this tax increase [14], to date, no study has examined whether smokers perceive cigarette tax increases to be a helpful vehicle to modify their smoking behaviors, and whether these perceptions are associated with subsequent smoking cessation behaviors. We used data collected through the Minnesota Adult Tobacco Survey (MATS) Cohort Study to fill this knowledge gap. As a result of the 2009 federal tobacco tax increase, the average retail price for a pack of cigarettes in Minnesota in 2009 was $4.81, slightly higher than the US average [13]. Minnesota was ranked 22nd in state cigarette excise taxes at the time [15]. Similar to US adults as a whole, the prevalence of smoking among Minnesota adults declined between 1999 and 2010 [16]. In 2010, the prevalence of smoking was 19.9% in the US and 16.1% in Minnesota [16]. The first research objective of this study was to examine Minnesota smokers who perceived the 2009 federal cigarette tax increase to be helpful in promoting a change in their smoking cessation behaviors. An additional objective was to assess the associations between perceiving the tax increase to be helpful in promoting smoking cessation and subsequent smoking cessation behaviors.

## Methods

### Study population

The Minnesota Adult Tobacco Survey (MATS) Cohort Study was designed to further the understanding of the quitting process and the influence of various factors (e.g., tobacco control policies and media campaigns) on smoking related attitudes and behaviors. The details of the study are reported elsewhere [17]. In brief, participants of the cohort were drawn from the 12,580 MATS 2007 participants who were randomly selected from the Minnesota adult population (n = 7,532) and Blue Cross Blue Shield of Minnesota membership (n = 5,048). Those who had smoked more than 100 cigarettes in their lifetime, and were current smokers or former smokers who quit smoking less than 10 years prior to 2007 (n = 3,147) were eligible for the cohort. Among these, 2,436 (77%) agreed to participate and were interviewed every year between April and June from 2008 to 2010. Surveys were conducted by trained interviewers used computer-assisted telephone interviewing and participants received a $20 incentive after completing each survey. For this study, we included participants who reported smoking cigarettes at least one day in the past 30 days on the 2009 survey and those who reported quitting smoking after April 1, 2009 (baseline, total n = 727). Of those, 609 completed the 2010 survey (retention rate = 83.6%). When compared to current smokers who participated in the most recent statewide cross-sectional tobacco use survey (2010 Minnesota Adult Tobacco Survey), participants who completed the baseline survey were less likely to be between ages 20–29, less likely to have an income ≤ $35,000, less likely to have lower education, and smoked fewer days in the past 30 days. Participants who were lost to follow-up were more likely to be younger and lighter smokers at baseline (p < 0.05). All study protocols and instruments were reviewed and approved by the Westat Institutional Review Board.

### Measures

At baseline, the cigarette tax increase was described as follows: “In March of this year, a 61 cent cigarette tax increase took effect nationwide.” We then asked the participants, “Did it help you think about quitting?”, “Did it help you cut down on cigarettes?”, and “Did it help you make a quit attempt?” (yes/no). We also collected information on demographic variables at baseline. These included age, gender, education, and income. We assessed the respondents’ use of the following strategies to save money on cigarettes in the past year: buying a cheaper brand, rolling their own cigarettes, using another form of tobacco, using coupons/promotions, purchasing cartons instead of packs, and buying from less expensive places (yes/no) [18]. The total numbers of strategies used (0–6) represent the extent to which the participants used price-minimizing strategies.

At baseline and follow-up, participants reported the number of days they smoked in the past 30 days and the average number of cigarettes they smoked per day. We multiplied these two responses and divided by 20 (number of cigarettes in a pack) to calculate the number of packs of cigarettes smoked in the past 30 days at baseline and follow-up. Participants were classified as having cut down their cigarette consumption if they smoked fewer packs of cigarettes at follow-up than at baseline; otherwise participants were classified as not cutting down their cigarette consumption. At follow-up we also assessed whether they had stopped smoking for at least a day because they were trying to quit smoking (yes/no). Given that all participants were smokers at baseline, those who reported not smoking in the past 30 days at follow-up were classified as having quit smoking.

### Statistical analysis

We assessed the characteristics associated with the perceptions that the 2009 cigarette tax increase “helped think about quitting” using logistic regression models. We first included age, gender, education and income in the models to estimate the associations between these variables and the perceptions that the tax increase helped think about quitting. The number of cigarettes smoked per month (in quartiles) at baseline and the number of price-minimizing strategies used at baseline were then entered into the model. To avoid over-adjustment, only variables associated with the perceived helpfulness variables (p < 0.3) were included. The same analytic approach was used to examine the characteristics associated with the perceptions that the 2009 cigarette tax increase helped to cut down on cigarettes and helped make a quit attempt. Race/ethnicity was not included in the analysis because it was not associated with the perceived helpfulness variables (p > 0.3).

To assess the associations between the baseline perceptions that the tax increase helped them think about quitting and smoking cessation behaviors at follow-up, we first used a logistic regression model to generate a propensity score for agreeing that the tax increase helped to think about quitting, and included demographics, number of cigarettes smoked per month (in quartiles) and the use of price-minimizing strategies in the model. We then used three separate logistic regression models to assess the associations between the perception that the tax increase helped in thinking about quitting and (1) attempting to quit smoking, (2) cutting down cigarette consumption, and (3) smoking cessation at follow-up, and included the propensity score for agreeing that the tax increase helped think about quitting in these models. This approach allowed us to efficiently control for confounding due to the observed covariates [19,20]. The same approach was used to assess the association between perceiving the tax increase helped to cut down on cigarette consumption and helped to make a quit attempt at baseline and smoking cessation behaviors at follow-up. All analyses were conducted with SAS® version 9.2 [21].

## Results

The characteristics of the sample are summarized in Table 1. Overall, 64.6% (n = 467) of the sample reported that the 2009 tax increase helped them think about quitting, 46.7% (n = 338) reported that it helped them cut down on cigarettes, and 29.0% (n = 210) reported that it helped them make a quit attempt. Participants with lower education were more likely than those with a college education to report that the tax increase helped them think about quitting, helped them cut down on cigarettes, and helped them make a quit attempt (p < 0.05; Table 1). Participants with lower income were more likely than those earning ≥ $75,001 a year to report that the tax increase helped them cut down on cigarettes and helped them make a quit attempt (p < 0.05). Heavier smokers, after adjusting for demographics and the number of price-minimizing strategies used at baseline, were less likely than lighter smokers (smoked ≤ 6 packs in the past 30 days) to report that the tax increase helped them cut down on cigarettes and to make a quit attempt (p < 0.05). The number of price-minimizing strategies used was positively associated with reporting the tax increase helped in thinking about quitting, helped cut down on cigarettes, and helped to make a quit attempt (p < 0.05).

**Table 1:** Characteristics associated with perceiving the 2009 federal cigarette tax increase as helpful among Minnesota current smokers in 2009, the Minnesota Adult Tobacco Survey Cohort Study (n = 727)

	**Overall**	**Helped think about quitting**		**Helped cut down on cigarettes**		**Helped make a quit attempt**	
**Characteristics**	**N(%)**	**N(%)**	**AOR (95% CI)**	**N(%)**	**AOR (95% CI)**	**N(%)**	**AOR (95% CI)**
**Age**							
20–29	108 (14.8%)	61 (56.5%)	0.63 (0.37, 1.07)	43 (39.8%)	0.69 (0.41, 1.16)	28 (25.9%)	--
30–39	115 (15.8%)	69 (60.5%)	0.81 (0.48, 1.37)	45 (39.1%)	0.72 (0.43, 1.20)	33 (28.7%)	
40–49	154 (21.2%)	98 (63.6%)	0.88 (0.54, 1.44)	75 (48.7%)	0.97 (0.61, 1.56)	46 (29.9%)	
50–59	183 (25.2%)	131 (72.4%)	1.31 (0.80, 2.13)	98 (53.9%)	1.19 (0.75, 1.87)	52 (28.6%)	
60 or above	167 (23.0%)	108 (65.1%)	Ref.	77 (46.7%)	Ref.	51 (30.7%)	
**Gender**							
Male	349 (48.0%)	216 (62.3%)	0.82 (0.59, 1.13)	159 (45.6%)	--	104 (29.8%)	--
Female	378 (52.0%)	251 (66.8%)	Ref.	179 (47.7%)		106 (28.2%)	
**Education**							
High school/GED	292 (40.2%)	205 (70.7%)	**2.44 (1.57, 3.79)**	154 (52.7%)	**2.18 (1.40, 3.42)**	102 (34.9%)	**1.92 (1.17, 3.17)**
Some college	289 (39.8%)	186 (64.4%)	**1.82 (1.18, 2.80)**	137 (47.6%)	**1.85 (1.18, 2.98)**	78 (27.0%)	1.43 (0.86, 2.37)
College or above	145 (20.0%)	75 (52.5%)	Ref.	46 (32.2%)	Ref.	29 (20.3%)	Ref.
**Income**							
≤$35,000	246 (35.6%)	159 (65.2%)	1.03 (0.67, 1.58)	119 (48.4%)	1.47 (0.96, 2.24)	86 (35.0%)	**1.64 (1.04, 2.59)**
$35,001-$75,000	271 (39.2%)	180 (66.4%)	1.18 (0.78, 1.77)	143 (52.6%)	**1.85 (1.24, 2.77)**	77 (28.4%)	1.27 (0.81, 1.99)
≥$75,001	175 (25.3%)	101 (58.4%)	Ref.	60 (34.7%)	Ref.	39 (22.5%)	Ref.
**Number of cigarettes smoked per month in 2009**							
≤6 packs	182 (25.1%)	114 (63.3%)	--	105 (58.3%)	Ref.	80 (44.4%)	Ref.
7-15 packs	238 (32.8%)	155 (65.7%)		120 (50.6%)	**0.50 (0.32, 0.78)**	60 (25.2%)	**0.33 (0.21, 0.52)**
16-27 packs	130 (17.9%)	86 (66.2%)		58 (44.6%)	**0.40 (0.24, 0.66)**	25 (19.2%)	**0.21 (0.12, 0.37)**
≥28 packs	176 (24.2%)	111 (63.1%)		54 (30.7%)	**0.16 (0.10, 0.27)**	45 (25.6%)	**0.28 (0.17, 0.46)**
**Number of price minimizing strategies used in 2009 (0–6)**	1.6 (1.2)*	--	**1.11 (1.04, 1.38)**	--	**1.30 (1.12, 1.51)**	--	**1.25 (1.07, 1.47)**

Demographics are only adjusted for demographics in the column that the crude associations are significant (p < 0.3). Other variables are adjusted for significant demographics and other variables in the column (crude association p < 0.3). *Mean and standard deviation presented. Bolded estimates are statistically significant (p<0.05).

Among the 609 participants who completed the follow-up, 282 (47.1%) had attempted to quit smoking in the past 12 months, 244 (40.2%) cut back their cigarette consumption, and 77 (12.6%) reported not smoking in the past 30 days. Agreeing that the cigarette tax increase helped think about quitting, cut down on cigarettes, and make a quit attempt at baseline were significantly associated with an increased likelihood of making a quit attempt at follow-up (p < 0.05; Table 2). Our data also suggested positive associations between these perceptions and a reduction in cigarette consumption and smoking cessation, although these associations were not statistically significant (p > 0.05).

**Table 2:** Associations between perceived helpfulness of the 2009 federal cigarette tax increase at baseline and smoking cessation behaviors at follow-up among Minnesota current smokers in 2009, the Minnesota Adult Tobacco Survey Cohort Study (n = 609)

	**Cessation behaviors at follow-up (2010)**					
	**Made a quit attempt in the past 12 months**		**Cut down cigarette consumption in the past 12 months**		**Stopped smoking in the past 30 days**	
**Perceptions at baseline (2009)**	**N (%)**	**AOR (95% CI)**	**N (%)**	**AOR (95% CI)**	**N (%)**	**AOR (95% CI)**
**Helped think about quitting**						
Yes	213 (54.8%)	**2.58 (1.79, 3.73)**	161 (41.2%)	1.06 (0.74, 1.51)	52 (13.2%)	1.31 (0.76, 2.23)
No	68 (32.5%)	Ref.	83 (39.0%)	Ref.	23 (10.8%)	Ref.
**Helped cut down on cigarettes**						
Yes	162 (59.3%)	**2.52 (1.76, 3.61)**	120 (43.8%)	1.25 (0.88, 1.79)	41 (14.9%)	1.33 (0.79, 2.23)
No	119 (36.6%)	Ref.	124 (37.6%)	Ref.	34 (10.3%)	Ref.
**Helped make a quit attempt**						
Yes	124 (70.9%)	**3.80 (2.54, 5.69)**	74 (42.1%)	1.10 (0.75, 1.62)	26 (14.8%)	1.13 (0.65, 1.97)
No	158 (37.3%)	Ref.	170 (39.6%)	Ref.	49 (11.4%)	Ref.

Bolded estimates are statistically significant (p < 0.05). Estimates for each perception on each outcome were adjusted for the propensity score for endorsing the perception, which accounted for age, gender, education, income, smoking intensity (in quartiles), and number of price-minimizing strategies used at baseline.

## Discussion

We found that a significant proportion of Minnesota adult smokers thought that the 2009 federal cigarette tax increase was helpful in promoting smoking cessation. More importantly, those who perceived the tax increase as helpful were more likely than those who did not to subsequently attempt to quit smoking. Our findings represent an intersection between economic and cognitive processes. Cognitively, many smokers want to quit smoking but have not been successful in quitting on their own, and therefore they may look for commitment devices [12]. Our findings imply that a cigarette tax increase may trigger smokers to re-evaluate the financial burden of smoking. This re-evaluation may then lead smokers to see cigarette tax increases as opportunities for them to take action, as supported by previous research showing that cost of tobacco is a commonly cited reason and trigger for smoking cessation [5,7,22,23]. Thus, cigarette tax increases may represent an opportunity for a smoker to move from having low intention to quit smoking to taking action to quit smoking. Traditionally, media campaigns related to tobacco use have been either cessation-focused or generally anti-smoking [24]. Few, if any, of these media campaigns have specifically viewed a cigarette tax as a commitment opportunity. Given our findings, using media campaigns to promote cigarette tax increases as opportunities to quit smoking may encourage more smokers to make a quit attempt.

We found that smokers with lower education and lower income were generally more likely than those with a college education or higher income (annual income ≥ US$75,001) to think that the cigarette tax increase helped them think about quitting, to reduce cigarette consumption, and to make a quit attempt. This is consistent with earlier research that has found lower socioeconomic status (SES) smokers were more likely to report cost as a motive/trigger to smoking cessation [22,23]. Cigarette taxes are generally viewed as regressive because lower SES smokers pay a higher proportion of their income than higher SES smokers on the taxes [11,25]. However, cigarette tax increases could be progressive. Our findings suggest that compared to higher SES smokers, more lower SES smokers perceive cigarette tax increases as helpful in supporting their cessation behaviors, and they take the opportunity to commit to quit smoking. This may, in part, explain why lower SES smokers are more responsive than higher SES smokers to a cigarette tax increase [26-28], resulting in reduced social inequalities of tobacco use [9,10]. It is also encouraging to observe lower SES smokers to be more likely than higher SES smokers to perceive cigarette tax increases as helpful since smokers of lower SES smokers are usually heavier smokers [29], and therefore disproportionately affected by the harmful effect of cigarette smoking.

It is not surprising that, after adjusting for SES, heavier smokers were still less likely to perceive the cigarette tax increase as helpful in promoting smoking cessation. Specifically, the more someone smoked the less likely they were to perceived the tax increase as helpful in cutting down on cigarettes. Perhaps, the nicotine addiction and social environment of heavier smokers (e.g., having friends who smoke) makes it harder for them to think about changing their smoking behaviors. This is supported by previous studies that found heavier smokers were less likely to be successful in quitting [30]. However, it is noteworthy that even among the heaviest smokers who smoked ≥ 28 packs of cigarette per month, about 30% of them reported that the cigarette tax increase helped them cut down on cigarettes or to make a quit attempt. This implies that a cigarette tax increase is capable of motivating even the heaviest smokers to engage in the process of smoking cessation. Smokers who used price-minimizing strategies were more likely to perceive the tax increase as a stimulus to quitting, and even higher prices may be necessary to counteract the effectiveness of price-minimizing strategies and thereby support cessation.

Because of a relatively small sample size and the low prevalence of successful smoking cessation in our study, the positive association between perceived helpfulness of the cigarette tax increase in assisting smoking cessation and actual smoking reduction and cessation did not reach the level of statistical significance. Subsequent studies with larger sample sizes are needed to test this finding. Another limitation of the study is that the sample of smokers came from a single Midwestern state, which limits the generalizability of our findings to states with more racially/ethnically diverse populations. The differences between the cohort sample and a later statewide cross-sectional sample of smokers and attrition of the cohort also limit the external validity of our findings; however, the internal validity of the study should not be affected since we controlled for the variables associated with attrition.

## Conclusions

We found that a significant proportion of smokers in our statewide sample reported that the 2009 cigarette excise tax increase helped them think about quitting, cut down on cigarettes, and make a quit attempt, particularly among smokers of lower socio-economic status. Media campaigns to promote cigarette tax increases as a helpful device and an opportunity to quit smoking may further assist smokers to attempt to quit smoking.

## Consent

Participants provided active consent for their participation in this study.

## Competing interests

The authors declare that they have no competing interests.

## Authors’ contributions

KC and RB made substantial contributions to conception and design of the study. RB contributed to acquisition of data. KC and RB contributed substantially to analysis and interpretation of the data, drafting the manuscript, and provided final approval of the version to be published. Both authors read and approved the final manuscript.

## Pre-publication history

The pre-publication history for this paper can be accessed here:

http://www.biomedcentral.com/1471-2458/13/965/prepub
